# Adrenaline stimulates the proliferation and migration of mesenchymal stem cells towards the LPS-induced lung injury

**DOI:** 10.1111/jcmm.12283

**Published:** 2014-03-31

**Authors:** Xiaodan Wu, Zhiming Wang, Mengjia Qian, Lingyan Wang, Chunxue Bai, Xiangdong Wang

**Affiliations:** a Department of Pulmonary Medicine, Zhongshan Hospital, Fudan UniversityShanghai, China; b Department of Medical Oncology, Zhongshan Hospital, Fudan UniversityShanghai, China; c Biomedical Research Center, Zhongshan Hospital, Fudan UniversityShanghai, China

**Keywords:** acute lung injury, mesenchymal stem cells, lipopolysaccharide, inflammation, adrenergic receptor agonists

## Abstract

Bone marrow-derived mesenchymal stem cells (BMSCs) could modulate inflammation in experimental lung injury. On the other hand, adrenergic receptor agonists could increase DNA synthesis of stem cells. Therefore, we investigated the therapeutic role of adrenaline-stimulated BMSCs on lipopolysaccharide (LPS)-induced lung injury. BMSCs were cultured with adrenergic receptor agonists or antagonists. Suspensions of lung cells or sliced lung tissue from animals with or without LPS-induced injury were co-cultured with BMSCs. LPS-stimulated alveolar macrophages were co-cultured with BMSCs (with adrenaline stimulation or not) in Transwell for 6 hrs. A preliminary animal experiment was conducted to validate the findings in *ex vivo* study. We found that adrenaline at 10 μM enhanced proliferation of BMSCs through both α- and β-adrenergic receptors. Adrenaline promoted the migration of BMSCs towards LPS-injured lung cells or lung tissue. Adrenaline-stimulated BMSCs decreased the inflammation of LPS-stimulated macrophages, probably through the expression and secretion of several paracrine factors. Adrenaline reduced the extent of injury in LPS-injured rats. Our data indicate that adrenaline-stimulated BMSCs might contribute to the prevention from acute lung injury through the activation of adrenergic receptors, promotion of proliferation and migration towards injured lung, and modulation of inflammation.

## Introduction

Acute lung injury and acute respiratory distress syndrome are major causes of acute respiratory failure in critically ill patients, responsible for more than 40% mortality [1–3], even though there are significant improvements in supportive care. Previous studies demonstrated that bone marrow-derived mesenchymal stem cells (BMSCs) could attenuate experimental acute lung injury induced by lipopolysaccharide (LPS) [4,5], bleomycin [6], ventilation [7,8] or ischaemia-reperfusion [9]. Bone marrow-derived mesenchymal stem cells could be engrafted as type I and II epithelial cells or endothelial cells in the injured lung for the repair [6,10]. Intratracheal administration of BMSCs could prevent LPS-induced acute lung injury and improve the survival in non-bone marrow-suppressed mice [11]. Bone marrow-derived mesenchymal stem cells were also found to modulate the inflammation and reduce lung injury through the release of paracrine factors [11–14]. Bone marrow-derived mesenchymal stem cells could attenuate sepsis by releasing prostaglandin E2, which reprogrammed macrophages to increase interleukin (IL)-10 production [12]. Interleukin-1 receptor antagonist (IL-1ra) could also mediate the anti-inflammatory and anti-fibrotic effect of BMSCs during bleomycin-induced lung injury [13].

Adrenergic receptor agonists, especially β-adrenergic agonists, have the potential role in the treatment of acute lung injury by accelerating alveolar fluid reabsorption [15,16] and suppressing inflammation [17]. Those agonists could increase DNA synthesis of embryonic stem cells [18] and BMSCs [19], and were involved in the protective role against oxidative stresses in mesenchymal stem cells [20]. The present study aims to investigate the therapeutic role of adrenergic receptor agonists and antagonists in biological behaviours of BMSCs, including α- and β-adrenergic receptor agonist adrenaline, α-agonist norepinephrine, α-antagonist phentolamine, β-agonist isoproterenol or β-antagonist propranolol. We also studied the role of adrenaline in migration of BMSCs in *ex vivo* models of LPS-induced lung injury, and explored whether adrenaline could help BMSCs modulate inflammation through release of paracrine cytokines. Therapeutic roles of adrenaline-stimulated BMSCs in acute lung injury were furthermore investigated in an experimental model of acute lung injury induced by the intratracheal instillation of LPS.

## Materials and methods

### Animals

A total of 66 male Sprague-Dawley rats, weighing 200–250 g, were obtained from the Animal Center of Fudan University, Shanghai, China, and used in the experiment. All study protocols were approved by the Animal Care Committees of Fudan University. All animal experiments were conducted in accordance with the Guide for the Care and Use of Laboratory Animals published by the National Academy of Sciences [21].

### Materials

Adrenaline, norepinephrine, isoproterenol, phentolamine and propranolol were purchased from Sigma-Aldrich (St. Louis, Mo, USA). CM-dil was from Molecular Probes (CA., USA). ELISA kits to measure tumour necrosis factor-α (TNF-α), IL-1β, IL-6, IL-10, IL-13, angiopoietin-1, keratinocyte growth factor (KGF) and IL-1ra were purchased from R&D Systems (Shanghai, China). QuantiTect SYBR Green RT-PCR kit was purchased from QIAGEN (CA, USA). Primary antibodies against IL-10 and IL-1ra for Western blot were purchased from Santa Cruz Biotechnology (CA, USA) and against IL-13 and KGF were from Invitrogen (CA, USA).

### Cell culture

BMSCs were isolated from rat bone marrow as described previously [22,23]. In brief, the bone marrow was flushed from the tibia and femur of rats with DMEM medium (Sigma) containing 5% foetal calf serum (FCS, Invitrogen) plus penicillin and streptomycin at 100 U/ml and 0.1 mg/ml, separately (Invitrogen) and then filtered through a sterile filter with the 100 μm pore to produce a single cell suspension. The bone marrow cells were plated in culture dishes with DMEM plus 20% FCS with antibiotics and allowed to adhere for 24 hrs at 37°C with 5% CO_2_. Non-adherent cells were then removed. Medium was replaced twice weekly and cells were subcultivated at a one-to-three split ratio by trypsinization at 0.25% trypsin and 1 mM EDTA. Immunophenotypes of BMSCs were determined by flow cytometry to prove the purity of BMSCs and differentiation potential was demonstrated by adipogenesis and osteogenesis assays. The 3^rd^ to 6^th^ passages of cells were utilized for experiments.

Alveolar macrophages were isolated from rats by bronchoalveolar lavage using 5 ml PBS for four times. Cells were centrifuged at 100 × *g* for 6 min. and suspended in RPMI 1640 containing 5% FCS and 1% penicillin/streptomycin at a concentration of 5 × 10^4^ cells/ml. Cells were then incubated at 37°C with 5% CO_2_, 2 hrs after which non-adherent cells were discarded and adherent cells were washed gently with RPMI 1640. Tissue cells were isolated from the lung with the tissue mechanically macerated to create a suspension and placed in ice-cold PBS in a 100-mm cell culture plate. The cells were pelleted, washed twice with DMEM and then re-suspended at a concentration of 2 × 10^6^ cells/ml.

### Alive measurement of cell biological behaviours

The cell biological behaviours including the cell proliferation, division, death and movement were measured by the real-time cell monitoring system, using a Cell-IQ cell culturing platform (Chip-Man Technologies, Tampere, Finland), equipped with a phase-contrast microscope and a camera. The equipment was controlled by Imagen software (Chip-Man Technologies). Images were captured at 5 min. intervals for 72 hrs and analyses were carried out with freely distributed Image software (McMaster Biophotonics Facility, Hamilton, USA). BMSCs were stimulated with PBS or adrenaline at concentrations of 0–100 μM, 24 hrs after cell culture at the density of 10^4^/ml in a 24-well plate. Each group contained eight replicate image sites. Increased percentages of numbers of total cells, divided cells, dead cells and cell movement length were measured and calculated. BMSCs were also stimulated with α-adrenergic receptor agonist (norepinephrine), β-adrenergic receptor agonist (isoproterenol) or in combination of adrenaline with phentolamine or propranolol, respectively, to compare with cells stimulated with vehicle or adrenaline alone.

### Cell migration

Lung cells from animals with the intratracheal administration of vehicle or LPS (5 mg/kg, Sigma) for 24 hrs were seeded in a 24-well plate at a density of 2 × 10^6^ cells/ml. BMSCs from normal rats were placed in filters (Millipore, Billerica, MA) with 8 μm size pore at a density of 5 × 10^4^/ml. Lung cells were located in the lower compartment of the Transwell system, while BMSCs in the upper, as shown in Figure S1A, where adrenaline at 10 μM was added. The migration of BMSCs from the upper compartment to the lower was observed by Giemsa staining (Ximeijie, Beijing, China), 36 hrs after the co-culture. Lung tissues obtained from rats 24 hrs after the intratracheal administration of LPS or vehicle were sliced to 2 × 2×2 mm^3^ pieces and then co-cultured with CM-dil labelled BMSCs in the 6-well plate for 24 hrs, with vehicle or adrenaline stimulation (Figure S1D). Migration towards and adhesion of BMSCs to lung tissue were observed by fluorescence microscope. Each group contained six replicate image sites.

### Enzyme-linked immunosorbent assay (ELISA)

Alveolar macrophages stimulated with LPS at 4 μM were co-cultured with or without BMSCs, which were stimulated with vehicle or adrenaline at 10 μM for 24 hrs. In a Transwell system with the separation of 0.4 μm size pore, BMSCs in the upper compartment were co-cultured with macrophages in the lower for 6 hrs (Figure S1B). The cell culture supernatant was then collected for the analyses of TNF-α, IL-1β, IL-6, IL-10 and IL-13 by ELISA according to manufacturer's instructions. On the other hand, BMSCs stimulated with vehicle or adrenaline at 10 μM for 24 hrs were co-cultured with macrophages stimulated with vehicle or LPS at 4 μM in the Transwell system. Macrophages in the upper compartment were co-cultured with BMSCs in the lower for 6 hrs (Fig. S1C). The cell culture supernatant was then collected for the measurements of angiopoietin-1, KGF and IL-1ra by ELISA. Cells were collected for the following PCR and Western blot analysis.

### Real-time quantitative PCR analysis

Total RNA was extracted from cells using Trizol (Invitrogen) according to the manufacturer's protocol. Quantitative real-time RT-PCR detection was performed with the QuantiTect SYBR Green RT-PCR kit, and RNA was reversely transcribed into cDNA according to the manufacturer's instructions. The PCR reaction was followed by melting curve analysis to confirm the specificity and identity of the RT-PCR products as listed in Table 1.

**Table 1:** PCR primer sets

Genes	Sense primers (5′–3′)	Antisense primers (5′–3′)
Angiopoitein-1	GAG TGA GGC AAG AGG TGT AG	ATC CAA GGT AGA AAG AGA CCA
KGF	GGG GTG GAA AGT GAA TAC TA	GCA GAG GTG TTG TAA TGG TT
IL-1ra	GAA AAT AGG CGG TAG GCT	AAG TGG TGG GGA GAT TAT G
IL-10	GCT ATG TTG CCT GCT CTT AC	TGA CTG GGA AGT GGG TG
IL-13	CTT GCC TTG GTG GTC TTG	TGG TCT TGT GTG ATG TTG CT

Primer pairs were used to detect and confirm the expression of angiopoitein-1, KGF, IL-1ra, IL-10 and IL-13 in BMSCs. BMSCs, bone marrow-derived mesenchymal stem cells; KGF, keratinocyte growth factor; IL-1ra, interleukin-1 receptor antagonist; IL-10, interleukin-10; IL-13, interleukin-13.

### Western blot

Intracellular proteins were extracted from BMSCs by RIPA lysis immediately. Protein samples (50 μg) were mixed with an equal volume of 5 × SDS sample buffer, boiled for 5 min., and then separated through 10% SDS-PAGE gels. After electrophoresis, proteins were transferred to PVDF membranes by electrophoretic transfer. Membranes were blocked in 5% dry milk (1 hr), rinsed and incubated with primary antibodies (diluted at 1:1000 or 1:2000) in Tris-buffered saline (TBS) at 4°C overnight. Primary antibody was then removed by washing in TBS thrice, and labelled by incubating with peroxidase-labelled secondary antibodies of 0.1 mg/ml against mouse or rabbit for 1 hr. Bands were washed thrice in TBS and visualized by ECL luminous liquid and exposed to X-ray film. Intracellular levels of IL-10, IL-13, KGF or IL-1ra were measured to evaluate potential effects of adrenaline and LPS on cytokine production of BMSCs. All results were calculated by Phoretix 1D software and expressed as ratios to β-actin protein as the loading control.

### *In vivo* experiment

Animals were pre-treated intravenously with BMSCs, adrenaline-stimulated BMSCs, conditioned medium of BMSCs, adrenaline or vehicle 24 hrs before the intratracheal instillation of LPS at 5 mg/kg or the same volume of PBS. Histological changes were determined 24 hrs after LPS or vehicle instillation. Rats were randomly divided into seven groups (*n* = 8 for each group): A) animals pre-treated with vehicle and challenged with PBS; B) animals pre-treated with vehicle and challenged with LPS; C) animals pre-treated with 5 × 10^6^ BMSCs in 0.5 ml PBS and challenged with LPS; D) animals pre-treated with adrenaline (10 μM)-stimulated BMSCs (5 × 10^6^) and challenged with LPS; E) animals pre-treated with 0.5 ml conditional medium of BMSCs and challenged with LPS; F) animals pre-treated with 0.5 ml conditional medium from adrenaline-activated BMSCs and challenged with LPS or G) animals pre-treated with 0.5 ml adrenaline at 10 μM and challenged with LPS. After exsanguination, the lungs were cut and stored in 4% paraformaldehyde. After 5 days, lung samples were inflated, fixed in formalin, paraffin-embedded and sectioned for histological analysis. Slides were washed and counterstained with haematoxylin and eosin.

### Statistics

Data are presented as mean ± SD and analysed using SAS 6.12 software (SAS Institute, Cary, NC, USA). Comparisons among multiple groups were performed with one-way anova. Least significant difference method was used to compare between two groups. *P* < 0.05 was considered statistically significant.

## Results

### Bone marrow-derived mesenchymal stem cells (BMSCs)

**Fig. 1:**
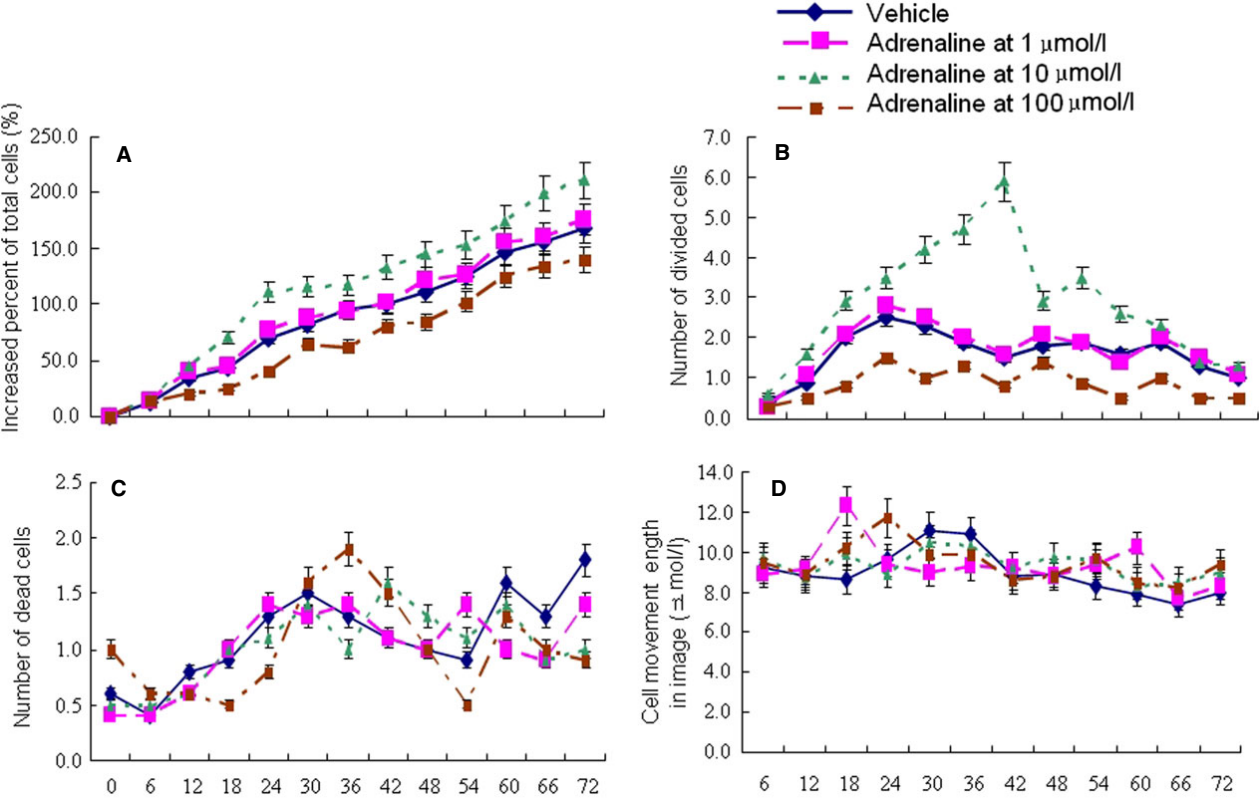
Cell biological behaviours of BMSCs after stimulation with adrenaline at 0–100 μM. BMSCs were stimulated with vehicle or adrenaline at 0–100 μM. Increased percentage of total cell number, divided cell number, dead cell number and cell movement length of BMSCs were measured by Cell-IQ Alive Image Monitoring System for 72 hrs. Data were presented as mean ± SD and each group had eight measurements. Abbreviations: BMSCs, bone marrow-derived mesenchymal stem cells.

**Fig. 2:**
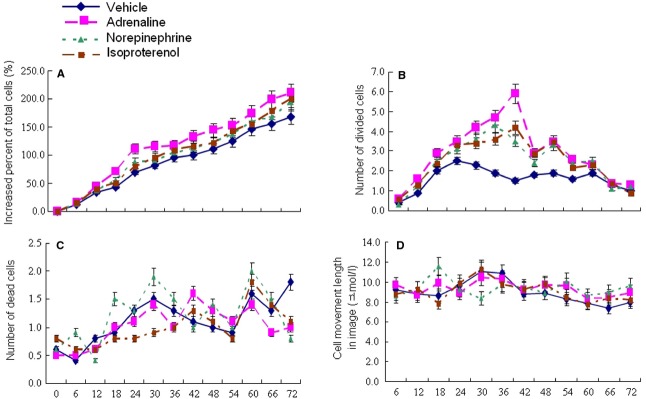
Cell biological behaviours of BMSCs after stimulation with different adrenergic receptor agonists. BMSCs were stimulated with vehicle, adrenaline at 10 μM, norepinephrine at 10 μM or isoproterenol at 10 μM respectively. Increased percentage of total cell number, divided cell number, dead cell number and cell movement length of BMSCs were measured by Cell-IQ for 72 hrs. Data were presented as mean ± SD and each group had eight measurements.

**Fig. 3:**
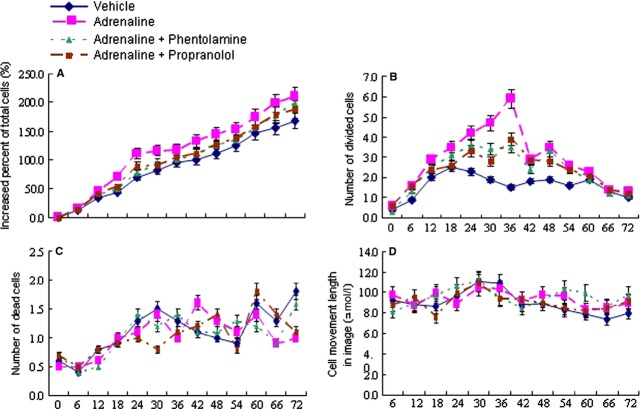
Cell biological behaviours of BMSCs after stimulation with different adrenergic receptor antagonists. BMSCs were stimulated with vehicle, adrenaline at 10 μM, adrenaline combined with phentolamine at 10 μM or adrenaline combined with propranolol at 10 μM, respectively. Increased percentage of total cell number, divided cell number, dead cell number and cell movement length of BMSCs were measured by Cell-IQ for 72 hrs. Data were presented as mean ± SD and each group had eight measurements.

BMSCs at passages 3–6 had a spindled, fibroblast appearance in culture, and the cells were 99.83% positive for CD29, 97.87% positive for CD44, 3.12% positive for CD34. Differentiation assays demonstrated that BMSCs retained their ability to form osteoblasts and adipocytes. Total number of BMSCs or divided cells significantly increased from 6 hrs and reached the peak at 18–24 hrs or at 36 hrs, respectively, after the stimulation of adrenaline at 10 μM, as compared to control (*P* < 0.05, Fig. 1A and B). Adrenaline at 1 μM did not affect proliferation, while adrenaline at 100 μM decreased it. Adrenaline at different concentrations did not influence dead cell number and cell movement (Fig. 1C and D). As a result, 10 μM was chosen as the concentration used in our study. Norepinephrine and isoproterenol had similar stimulatory effects on proliferation of BMSCs, but effects were relatively weaker than adrenaline. The two adrenergic agonists- norepinephrine and isoproterenol did not affect cell death and movement (Fig. 2 A–D). Phentolamine and propranolol had similar inhibitory effects on proliferation of BMSCs when used in combination with adrenaline. The two adrenergic antagonists did not affect dead cell number and cell movement (Fig. 3 A–D).

### Cell migration

**Fig. 4:**
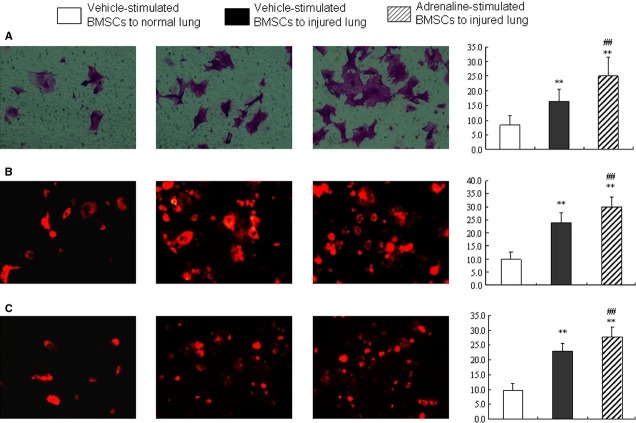
Migration of BMSCs towards lung cells or lung tissue. (**A**) Suspensions of lung cells from normal rats or from LPS-injured rats were co-cultured with BMSCs. Adrenaline at 10 μM was added to the Transwell system. Giemsa staining of the upper wells were observed after 36 hrs in co-culture. The first three figures showed BMSCs' migration towards normal lung cells, BMSCs' migration towards LPS-injured lung cells and migration of adrenaline-stimulated BMSCs towards LPS-injured lung cells, respectively (magnification 200 × ). Migration of BMSCs towards lung cells was quantified by counting the Giemsa staining cells per high-power field and illustrated in the bar graph. Values are the mean ± SD in three separate experiments; *n* = 6 in each group. **P < 0.01 significantly different from the first group, ^##^ *P* < 0.01 significantly different from the second group. (**B** and **C**) Lung tissue was obtained from normal rats or from LPS-injured rats and was sliced to blocks, which were co-cultured with CM-dil labelled BMSCs and adrenaline for 24 hrs. BMSCs' migration (**B**) towards and adhesion (**C**) to lung tissue were observed by fluorescence microscope (magnification 200 × ). Migration and adhesion of BMSCs were quantified by counting the labelled cells per high-power field and shown in bar graph. Values are the mean ± SD in three separate experiments; *n* = 6 in each group. **P < 0.01 significantly different from the first group, ^##^*P* < 0.01 significantly different from the second group.

LPS-injured lung cells promoted migration of BMSCs, compared to normal lung cells (*P* < 0.01). Adrenaline at 10 μM further promoted BMSCs' migration towards LPS-injured lung cells, compared to normal lung or injury lung without adrenaline activation (*P* < 0.01; Fig. 4A). LPS-injured lung tissue promoted BMSCs' migration towards and adhesion to lung tissue (*P* < 0.01). Adrenaline at 10 μM further increased migration and adhesion of BMSCs to LPS-injured lung tissue, as compared to normal lung or injury lung without adrenaline stimulation (*P* < 0.01; Fig. 4B and C).

### Levels of cytokines in co-culture supernatant

**Fig. 5:**
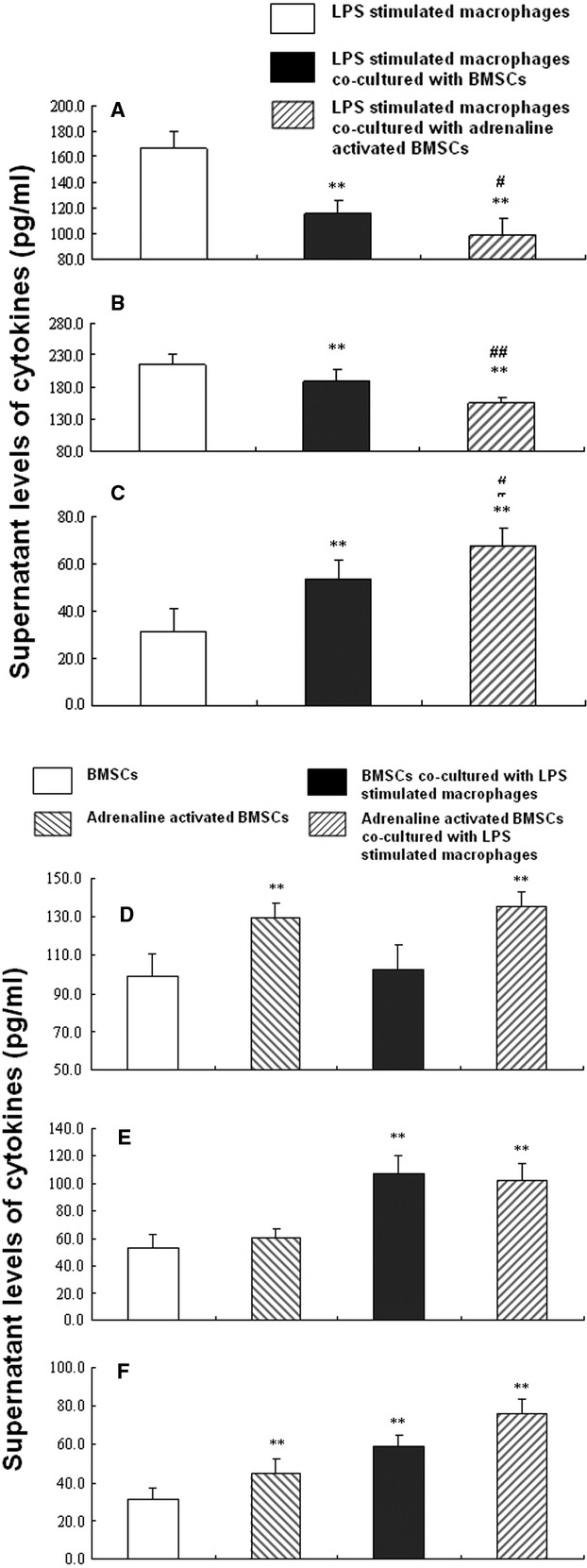
Supernatant cytokines concentrations. LPS-stimulated macrophages were cultured alone or with BMSCs, or with adrenaline-stimulated BMSCs in a Transwell system for 6 hrs. The cell culture supernatant was then collected for cytokine analysis by ELISA. Supernatant levels of TNF-α, IL-1β and IL-10 were shown in **A**–**C** respectively. Data are expressed as mean ± SD in three separate experiments; *n* = 6 wells per condition. **P < 0.01 significantly different from the first group; ^#^*P* < 0.05 significantly different from the second group; ^##^*P* < 0.01 significantly different from the second group. BMSCs or adrenaline-stimulated BMSCs were cultured alone or co-cultured with LPS-stimulated macrophages in a Transwell system for 6 hrs. The cell culture supernatant was then collected for analysis of Ang-1, KGF and IL-1ra by ELISA, which were shown in **D**–**F** respectively. Data are expressed as mean ± SD in three separate experiments; *n* = 6 wells per condition. **P < 0.01 significantly different from the first group. Abbreviations: BMSCs, bone marrow-derived mesenchymal stem cells; LPS, lipopolysaccharide; TNF-α, tumour necrosis factor-α; IL-1β, interleukin-1β; IL-10, interleukin-10. Ang-1, angiopoietin-1; KGF, keratinocyte growth factor; IL-1ra, interleukin-1 receptor antagonist.

BMSCs could modulate LPS-induced inflammation by significantly decreasing TNF-α (Fig. 5A) and IL-1β (Fig. 5B) or increasing IL-10 (Fig. 5C), as compared to control macrophages stimulated with LPS (*P* < 0.01). Adrenaline at 10 μM could enhance this effect by further decreasing TNF-α and IL-1β(*P* < 0.05 and 0.01, respectively) or increasing IL-10 (*P* < 0.05), as compared to BMSCs without adrenaline stimulation (Fig. 5 A–C). Co-culture with BMSCs or stimulation with adrenaline did not alter concentrations of IL-6 and IL-13 in supernatant. Adrenaline could increase the secretion of angiopoietin-1 and IL-1ra from BMSCs, as compared to BMSCs without adrenaline stimulation (*P* < 0.01), while LPS-stimulated macrophages could increase concentrations of KGF and IL-1ra in supernatant (*P* < 0.01). The combination of adrenaline and LPS-stimulated macrophages could significantly increase the secretion of angiopoietin-1, KGF or IL-1ra from BMSCs (*P* < 0.01; Fig. 5 D–F).

### mRNA expression levels of cytokines from BMSCs

**Fig. 6:**
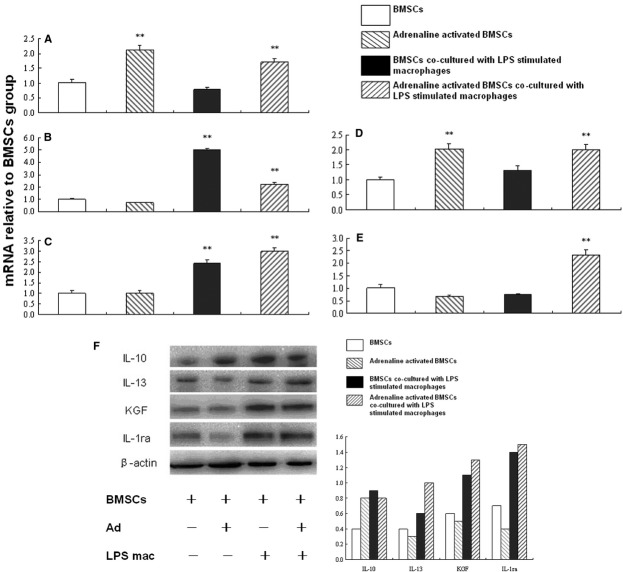
mRNA and protein expression levels of cytokines in BMSCs. BMSCs or adrenaline-stimulated BMSCs were cultured alone or co-cultured with LPS-stimulated macrophages in a Transwell system for 6 hrs. BMSCs were collected for Real-time quantitative PCR analysis of Ang-1, KGF, IL-1ra, IL-10 and IL-13, which was illustrated in **A**–**E** respectively. Data are expressed as mean ± SD in three separate experiments; *n* = 6 wells per condition. **P < 0.01 significantly different from the first group. (**F**) BMSCs were also collected for Western blot analysis of IL-10, IL-13, KGF and IL-1ra. Data are expressed as ratios to β-actin protein (loading control) in three separate experiments. Abbreviations: BMSCs, bone marrow-derived mesenchymal stem cells; LPS, lipopolysaccharide; Ang-1, angiopoietin-1; IL-10, interleukin-10; IL-13, interleukin-13; KGF, keratinocyte growth factor; IL-1ra, interleukin-1 receptor antagonist.

Adrenaline could significantly increase mRNA expression levels of angiopoitein-1 and IL-10 in BMSCs (*P* < 0. 01). Co-culture with LPS-stimulated macrophages could increase KGF and IL-1ra mRNA expression levels (*P* < 0.01). The combination of adrenaline and LPS-stimulated macrophages significantly increased mRNA expression levels of angiopoitein-1, KGF, IL-1ra, IL-10 and IL-13 (*P* < 0.01; Fig. 6A–E).

### Intracellular protein expression levels

Western blot analysis demonstrated significantly increased protein expression of IL-10 in BMSCs after the stimulation with adrenaline, IL-10, KGF and IL-1ra after co-culture with LPS-stimulated macrophages, or IL-10, IL-13, KGF and IL-1ra after the combination of adrenaline and LPS-stimulated macrophages (Fig. 6F).

### Histological changes of rat lung

**Fig. 7:**
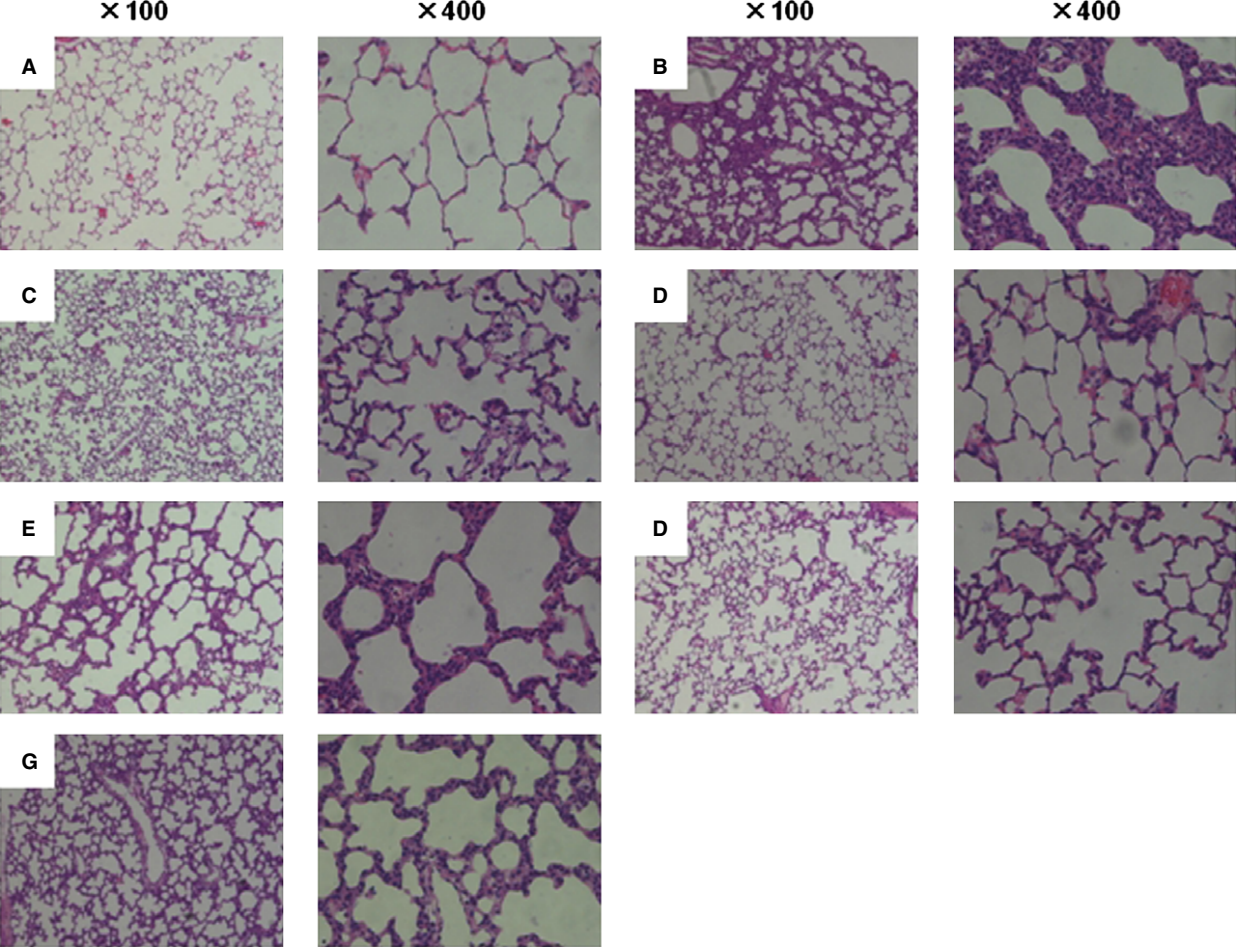
Lung histology by haematoxylin and eosin staining (magnification 100 × and 400 × ). Rats were randomly divided into seven groups (*n* = 8 for each group); (**A**) animals pre-treated with vehicle and challenged with PBS; (**B**) animals pre-treated with vehicle and challenged with LPS; (**C**) animals pre-treated with BMSCs and challenged with LPS; (**D**) animals pre-treated with adrenaline-stimulated BMSCs and challenged with LPS; (**E**) animals pre-treated with conditional medium of BMSCs and challenged with LPS; (**F**) animals pre-treated with conditional medium from adrenaline-activated BMSCs and challenged with LPS or (**G**) animals pre-treated with adrenaline and challenged with LPS. After exsanguinations, the lung was cut, fixed in formalin, paraffin-embedded and sectioned for histological analysis.

Transplantation with BMSCs or adrenaline-stimulated BMSCs decreased the extent of oedema, inflammation and haemorrhage induced by the intratracheal injection of LPS, while supernatant from BMSCs with or without adrenaline stimulation had less evident protective effects on LPS-induced lung injury. Adrenaline alone also had a slight therapeutic effects on reducing lung injury, as shown in Figure 7.

## Discussion

In this study, we found that: 1) adrenaline at 10 μM promoted proliferation of BMSCs through α- and β-adrenergic receptors; 2) adrenaline promoted migration of BMSCs towards injured lung cells or lung tissue; 3) adrenaline helped modulate inflammation by shifting from a pro-inflammatory to an anti-inflammatory response, which might result from some paracrine factors; 4) BMSCs with adrenaline stimulation could reduce LPS-induced histological injury in rats.

Kim *et al*. [18] found that adrenaline (epinephrine) increased DNA synthesis of mouse embryonic stem cells in a dose- and time-dependent manner with peak effect at 8 hrs and maximal effect at 1 μM, which was blocked by non-selective α-adrenergic antagonist and β-adrenergic antagonist. Similarly, Han *et al*. [19] demonstrated that norepinephrine-related α1-adrenergic receptor activation increased DNA synthesis of rat BMSCs in a dose- and time-dependent manner with peak effect at 8 hrs and maximal effect at 10 μM, which was blocked by phentolamine. We found that adrenaline promoted proliferation of BMSCs in a dose -dependent manner with maximal stimulatory effect at 10 μM and through both α- and β-adrenergic receptors.

To evaluate the effects of adrenaline on migration of BMSCs, we conducted two separate experiments. Firstly, we measured the effects of cell suspensions prepared from LPS-injured lung on the migration of BMSCs with adrenaline stimulation or not. When the lower compartment of Transwell contained cells from uninjured lung, no evidence of migration of BMSCs was found. However, when cells from LPS-injured lung were in the lower well, numerous BMSCs migrated towards the lower well. We speculated that cells from LPS-injured lung produced factors that stimulated BMSCs to migrate into the lung. This is consistent with the experiment conducted by Xu *et al*. [4], who also did *ex vivo* co-cultures of BMSCs and lung cells from endotoxaemic mice. We have noticed that adrenaline did not influence cell movement in the Cell-IQ analysis, but why could adrenaline increase the migration of BMSCs towards injured lung? We speculated that there might be two possible reasons: adrenaline stimulated proliferation of BMSCs and thus caused the migration of more cells; the interaction of adrenaline-stimulated BMSCs and injured lung cells might cause BMSCs to produce more cytokines, which influenced the migration of BMSCs. In another separate experiment, we established an *ex vivo* model for lung tissue and BMSCs co-culture to imitate the *in vivo* state, which further confirmed the findings in the former experiment. Our result demonstrated for the first time that injured lung tissue stimulated BMSCs' migration and adhesion, which we thought resulted from humoural factors produced by injured lung. In addition, adrenaline promoted BMSCs' migration and adhesion to injured lung tissue. These two separate experiments support the hypothesis that adrenaline might help BMSCs migrate into injured lung and enhance the interaction between BMSCs and lung cells, thus help injury repair.

BMSCs were demonstrated by prior studies to have anti-inflammatory effects on lung injury. Gupta *et al*. [11] found that intrapulmonary delivery of BMSCs in mice mediated a down-regulation of pro-inflammatory responses to endotoxin by reducing TNF-α and macrophage inflammatory protein-2, while increasing the anti-inflammatory cytokine IL-10. Their *in vitro* co-culture studies of BMSCs with alveolar macrophages provided evidence that the anti-inflammatory effect of decreasing TNF-α was paracrine and not cell contact dependent. Similarly, Xu *et al*. [4] discovered that BMSCs administration decreased both the systemic and local inflammatory responses induced by endotoxin in mice. Their *in vitro* co-cultures of BMSCs and lung cells from endotoxaemic animals demonstrated that BMSCs suppressed pro-inflammatory cytokines (including IL-1β, IL-6, IL-12 and macrophage inflammatory protein-1α) production by lung cells. In our study, alveolar macrophages were chosen to be co-cultured with BMSCs because they are the initial innate immune cell in the lung that is exposed to LPS in this model and orchestrate the inflammatory response and recruitment of other cells, such as neutrophils [24]. We found that BMSCs reduced inflammation by decreasing levels of TNF-α and IL-1β and increasing IL-10 concentration, which was not cell contact dependent. This was possibly because of the humoural interaction of BMSCs and LPS-stimulated macrophages. In addition, adrenaline helped BMSCs modulate inflammation by further decreasing pro-inflammatory cytokines and increasing IL-10, the mechanism of which we speculated was increased production of paracrine factors from BMSCs with the stimulation of adrenaline.

Some investigators have reported that BMSCs have a remarkable ability to modulate the immune system, in part through the release of paracrine factors including some growth factors that also have repair properties [25]. Nemeth *et al*. [12] established a sepsis model of caecal ligation and puncture in mice and found that macrophages derived IL-10 was a key for the protective effects of BMSCs. Their results also suggested that BMSCs (activated by LPS or TNF-α) reprogrammed macrophages by releasing prostaglandin E2. Ortiz *et al*. [13] demonstrated that the release of IL-1ra by mesenchymal stem cells (MSCs) was responsible for the protective effect on bleomycin-induced lung injury in mice. Lee *et al*. [26] demonstrated that allogeneic human MSCs or conditioned medium restored alveolar epithelial fluid transport and lung fluid balance from endotoxin induced acute lung injury in an *ex vivo* perfused human lung preparation. Using siRNA knockdown of potential paracrine soluble factors, they demonstrated that approximately 80% of the beneficial effect could be attributed to KGF. The same group of authors also found that antiopoietin-1 released from human MSCs could restore alveolar epithelial permeability to normal in cultured human alveolar epithelial type II cells after an inflammatory insult [27]. In our co-culture study, BMSCs could release KGF and IL-1ra when co-cultured with LPS-stimulated macrophages and this was further confirmed by PCR and Western blot analysis. Adrenaline alone could increase the secretion, mRNA expression levels and protein expression levels of several cytokines including angiopoietin-1, IL-1ra, IL-10, although the result was not consistent among different methods. However, when adrenaline was applied with LPS-stimulated macrophages, the expression and secretion of a panel of potential cytokines significantly increased, implying that the interaction among adrenaline, LPS-stimulated macrophages and BMSCs could influence cytokines production by BMSCs.

Although our *in vivo* experiment was preliminary, it found presumably for the first time that adrenaline could help BMSCs reduce injury in rats, thus validating the findings in *ex vivo* study. To evaluate whether the protective role of BMSCs was because of their secreted cytokines or the effect of adrenaline, we added three extra groups using supernatant from BMSCs with or without adrenaline stimulation or adrenaline for intravenous injection and found that their protective effects were relatively weak. We speculated that the interaction between adrenaline-stimulated BMSCs and injured microenvironment accounted for the therapeutic effects in LPS-injured rat lungs.

There are limitations to this study. Further studies including siRNA knockdown will be needed to determine which factor played a central role in modulating inflammation. The mechanism of adrenaline in stimulating proliferation, promoting migration and helping modulate inflammation needs to be further clarified.

In summary, these findings are the first evidence that adrenaline could promote the proliferation and migration of BMSCs, and help modulate inflammation in LPS-injured *ex vivo* rat lung. *In vivo* model of acute lung injury further validated the therapeutic roles of adrenaline-stimulated BMSCs and raise the possibility of using adrenergic receptor agonists-stimulated BMSCs for patients with acute respiratory distress syndrome.

## Supporting information

**Figure S1**. Study protocol.
