# Supplementary figures and images for: Adrenaline stimulates the proliferation and migration of mesenchymal stem cells towards the LPS-induced lung injury
Source: J Cell Mol Med. 2014 Mar 31;18(8):1612–22. doi: 10.1111/jcmm.12283 (PMC4190907; doi:10.1111/jcmm.12283)

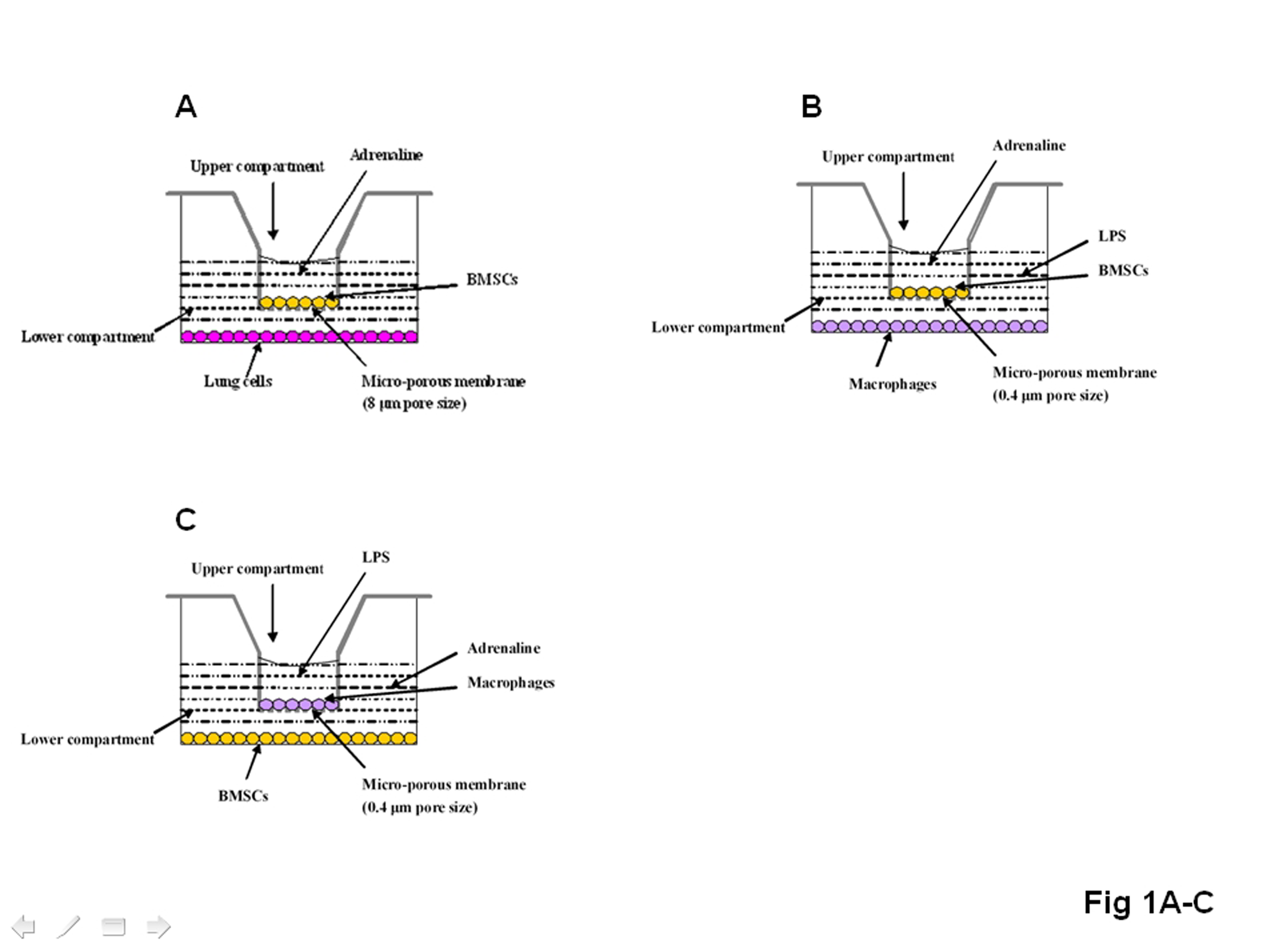

Supplement: Supplementary file 1 — Figure S1. Study protocol. [file jcmm0018-1612-SD1.tif]

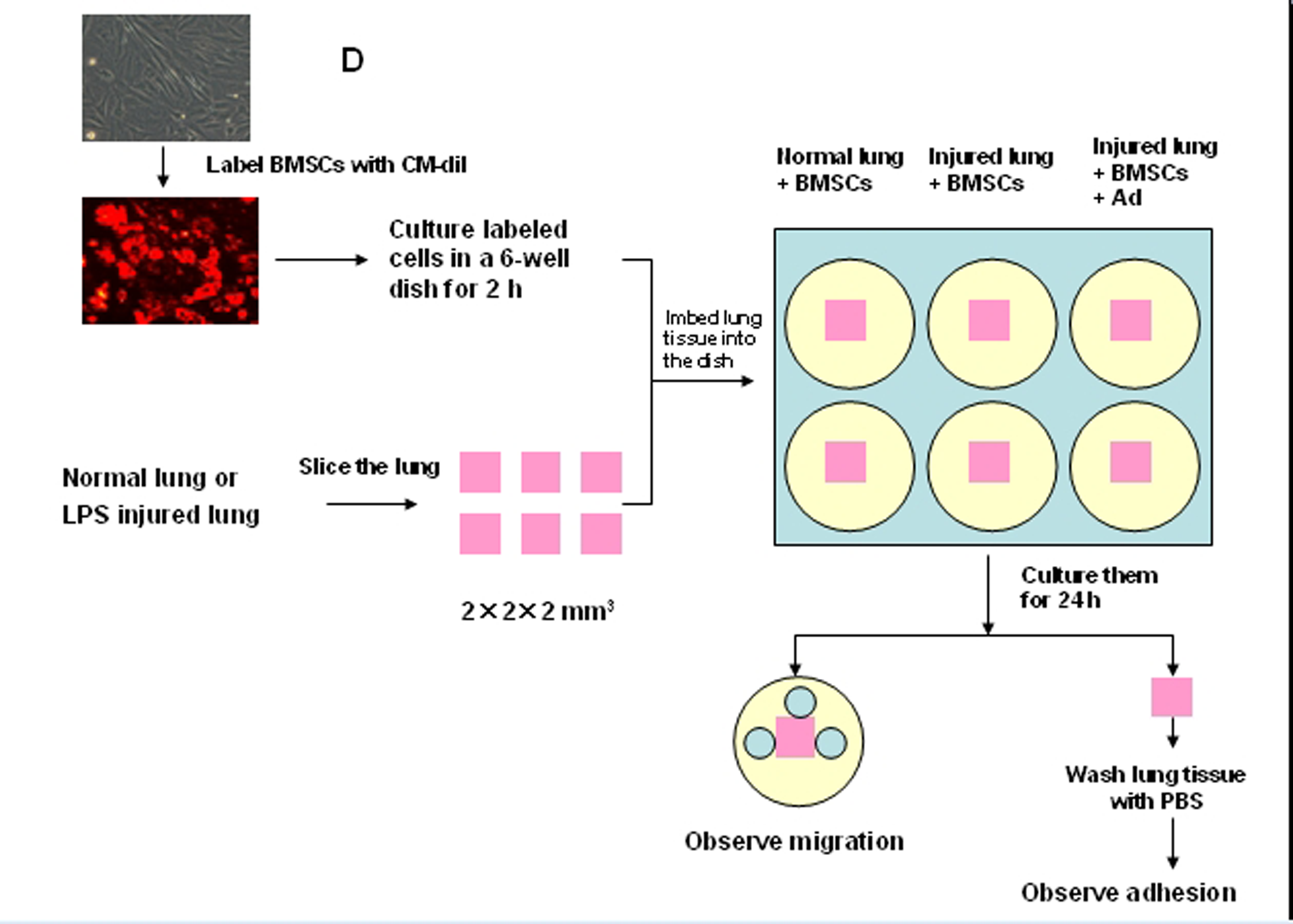

Supplement: Supplementary file 2 — Figure S1. Study protocol. [file jcmm0018-1612-SD2.tif]
